# Impact of Glycerol and Heating Rate on the Thermal Decomposition of PVA Films

**DOI:** 10.3390/polym17152095

**Published:** 2025-07-30

**Authors:** Ganna Kovtun, Teresa Cuberes

**Affiliations:** 1 Group of Nanotechnology and Materials, Mining and Industrial Engineering School of Almadén, University of Castilla-La Mancha, 13400 Almadén, Spain; 2 V.G. Baryakhtar Institute of Magnetism of the NAS of Ukraine, 03142 Kyiv, Ukraine

**Keywords:** poly(vinyl) alcohol films, PVA/glycerol blends, thermogravimetry, differential thermal analysis, deconvolution analysis, degradation rate, conversion, activation energy, isoconversional methods

## Abstract

This study analyzes the thermal degradation of PVA and PVA/glycerol films in air under varying heating rates. Thermogravimetric analysis (TGA) of pure PVA in both air and inert atmospheres confirmed that oxidative conditions significantly influence degradation, particularly at lower heating rates. For PVA/glycerol films in air, deconvolution of the differential thermogravimetry (DTG) curves during the main degradation stage revealed distinct peaks attributable to the degradation of glycerol, PVA/glycerol complexes, and PVA itself. Isoconversional methods showed that, for pure PVA in air, the apparent activation energy (Ea) increased with conversion, suggesting the simultaneous occurrence of multiple degradation mechanisms, including oxidative reactions, whose contribution changes over the course of the degradation process. In contrast, under an inert atmosphere, Ea remained nearly constant, consistent with degradation proceeding through a single dominant mechanism, or through multiple steps with similar kinetic parameters. For glycerol-plasticized films in air, Ea exhibited reduced dependence on conversion compared with that of pure PVA in air, with values similar to those of pure PVA under inert conditions. These results indicate that glycerol influences the oxidative degradation pathways in PVA films. These findings are relevant to high-temperature processing of PVA-based materials and to the design of thermal treatments—such as sterilization or pyrolysis—where control over degradation mechanisms is essential.

## 1. Introduction

Poly(vinyl alcohol) (PVA) is a synthetic polymer widely used in applications ranging from packaging [1,2] and biomedical materials [3] to coatings [4] and engineering [5], owing to its excellent mechanical properties, chemical resistance, and biodegradability. The incorporation of plasticizers, such as glycerol, into PVA has been widely employed to improve its flexibility and processability [6,7]. Recently, we carried out a thorough study on the influence of glycerol’s incorporation on the structural and physicochemical properties of thin PVA films [8]. From the analysis of the thermogravimetry (TGA) and differential thermal analysis (DTA) curves of the films for a heating rate of 25 °C/min, we investigated the thermal stability, the glass transition temperature, and the melting point and enthalpy of fusion of the pure PVA and PVA/glycerol films. Our data confirmed that the temperature at the onset of mass loss decreased with increasing glycerol content, and they let us identify a characteristic peak in the DTA curves related to the degradation of a glycerol-based phase at the second degradation stage, corresponding to the maximum weight loss, which degraded at a different temperature than pure PVA. In the current study, we have further explored the impact of glycerol’s incorporation on the thermal degradation behavior of PVA, considering different heating rates. While the role of glycerol as a plasticizer has already attracted considerable attention (see Kovtun et al. [8] and Refs. therein), its influence on PVA’s degradation behavior remains less understood. Glycerol can influence thermal stability and decomposition pathways by altering the activation energy profiles, potentially affecting both the degradation mechanism and the temperature range of thermal events. Understanding these effects is crucial for optimizing PVA-based materials for high-temperature applications.

Thermal analysis techniques, including TGA and DTA, are powerful tools for studying the degradation behavior of polymer systems [9]. These methods provide insights into key parameters like degradation onset temperatures, reaction rates, and additive effects. However, complex degradation mechanisms, such as those in PVA/glycerol systems, may involve overlapping processes that require deconvolution and kinetic investigation for a more detailed understanding. Kinetic analysis techniques like isoconversional methods [10,11,12,13] enable the estimation of activation energy across various conversion levels without assuming specific reaction models, making them particularly suited for studying multi-step reactions. Deconvolution of overlapping thermal events further aids in resolving the contributions of different degradation processes.

Here we present the results of the thermal degradation behavior of PVA/glycerol films in air at heating rates of 5 °C/min and 25 °C/min, comparing the results to those of pure PVA in air and in an inert atmosphere. Using TGA/differential thermogravimetric analysis (DTG) and mathematical deconvolution analysis combined with isoconversional methods for activation energy estimation, this work aims to examine the influence of glycerol on the degradation profile of PVA, including the identification of additional thermal events unique to PVA/glycerol systems, as well as to explore the relationship between heating rates, glycerol content, and degradation pathways and provide a kinetic evaluation of thermal degradation processes, focusing on activation energy’s dependency on conversion. Stepwise isothermal heating at selected temperatures was also carried out to gain further insight into the degradation process.

The findings contribute to a deeper understanding of the interplay between glycerol and PVA during thermal degradation. These insights are particularly relevant for high-temperature processing techniques, such as extrusion, melt blending, or thermal recycling. Furthermore, the knowledge gained from degradation kinetics can inform the design of thermal treatments, such as sterilization or pyrolysis protocols, ensuring safety, preserving material properties, and optimizing the composition of degradation products.

## 2. Materials and Methods

### 2.1. Materials and Sample Preparation

PVA (Mw 31,000–50,000 g/mol, 98–99% hydrolyzed) and glycerol (≥99.0%) were supplied by Merck (Darmstadt, Germany). The PVA was dissolved in distilled water with stirring at 90 °C and 400 rpm when heated in a water bath for 2 h, to prepare a 7.0 wt.% stock solution. Solutions of PVA with a 3.5 wt.% concentration, as well as mixed PVA/glycerol solutions containing 3.5 wt.% PVA and 1.0 wt.%, 2.0 wt.%, or 4.4 wt.% glycerol, were prepared from the 7.0 wt.% PVA stock solution, glycerol, and distilled water, before being stirred at 25 °C and 400 rpm for 20 min, poured on polystyrene Petri dishes, and evaporated at room temperature (20–25 °C) and ambient humidity (~50% R.H.). Films ~200 µm thick were obtained by this method (solution casting). The glycerol content in the prepared films in relation to the amount of dry PVA was 22% *w*/*w*, 36% *w*/*w*, and 55% *w*/*w*. The film preparation procedure is illustrated in Figure 1 of [8].

### 2.2. Thermogravimetric Analysis (TGA) and Differential Thermal Analysis (DTA)

The thermal behavior of the samples was examined with a Setaram model TG/DTA92 device (Caluire-et-Cuire, France), using a Pt crucible. Thermograms were recorded in an air atmosphere, within a temperature range from 20 °C to 600 °C, using heating rates of 5 °C/min, 15 °C/min, or 25 °C/min and an air flow rate of 33 mL/min. Thermograms for pure PVA films were additionally recorded in an argon atmosphere, within a temperature range from 20 °C to 600 °C, with a heating rate of 5 °C/min or 25 °C/min, and an argon flow rate of 41 mL/min. The data were analyzed using Originpro 2024 (Northampton, MA, USA) software. DTG was performed to identify the thermal transformation peaks, and to distinguish between two or more overlapping reactions. DTG curves were generated as the first derivative of weight with respect to temperature.

For the stepwise isothermal experiments, the films were heated at a rate of 5 °C/min and held for 30 min at each of the following temperatures: 50, 150, 200, 250, and 350 °C.

### 2.3. Kinetic Analysis

The reaction rate for solid-state decomposition is expressed by the following equation:

(1) $$\frac{d\alpha }{dt}=k\left(T\right)f\left(\alpha \right)=A\cdot exp\left(\frac{-E_{a}}{RT}\right)f(\alpha )$$

where α is a fraction decomposed at time t (the degree of conversion), k(T) is a temperature-dependent function, f(α) is a conversion function that depends on the mechanism of decomposition, A is the frequency factor, T is the absolute temperature, R is the gas constant (8.314 J mol^−1^ K^−1^), and Ea is the activation energy.

The degree of conversion is defined by the ratio of sample mass loss at a specific time to the total mass loss during the whole heating process:

(2) $$\alpha =\frac{m_{0}-m_{t}}{m_{0}-m_{f}}$$

where m_0_, m_f_, and m_t_ indicate the sample weight at the initial, final, and instant times, respectively.

For non-isothermal experimental conditions, a linear heating rate β (β = dT/dt) would be imported into Equation (1) to obtain the next equation:

(3) $$\frac{d\alpha }{dT}=\frac{A}{\beta }\cdot exp\left(\frac{-E_{a}}{RT}\right)f(\alpha )$$

Applying different theoretical analysis to Equation (3), the kinetic parameters can be calculated. The model-free or isoconversional methods allow for the independent determination of the activation energy without knowing the reaction mechanism. These methods are based on the idea that the rate constant (k) is only dependent on temperature for a reaction, and that the degree of conversion is constant. Isoconversional methods are categorized into differential methods, relying on approximating the so-called temperature integral and requiring data on T_α_(β) (for example, Friedman (FR)), and integral methods, using a determination of the reaction rate at an equivalent stage of the reaction for various heating rates (for example, Starink (STK), Flynn–Wall–Ozawa (FWO), and Kissinger–Akahira–Sunose (KAS)).

The methods used in this research to calculate the activation energy are discussed below.

Kissinger–kahira–Sunose (KAS) Method [10]:

(4) $$ln\left(\frac{\beta }{T^{2}}\right)=ln\left[\frac{AR}{E_{a}\cdot g(\alpha )}\right]-\frac{E_{a}}{RT}$$

where g(α) is the integral form of the conversion-dependent function f(α), which is dependent on the reaction mechanism.

The value of activation energy for each conversion is calculated from the slope of the linear plot of ln β/T^2^ versus 1/T.

Flynn–Wall–Ozawa (FWO) Method [12]:

(5) $$ln\left(\beta \right)=ln\left[\frac{AE_{a}}{R\cdot g(\alpha )}\right]-5.331-1.052\frac{E_{a}}{RT}$$

The value of activation energy for each conversion is calculated from the slope of the linear plot of ln(β) versus 1/T.

Starink (STK) Method [13]:

(6) $$ln\left(\frac{\beta }{T^{1.92}}\right)=ln\left[\frac{AE_{a}}{R\cdot g(\alpha )}\right]-1.0008\frac{E_{a}}{RT}$$

The value of activation energy for each conversion is calculated from the slope of the linear plot of ln(β/T^1.92^) versus 1/T.

Friedman (FR) Method [11]:

(7) $$\ln\left(\frac{d\alpha }{dt}\right)=\ln\left(A\cdot f\left(\alpha \right)\right)-\frac{E_{a}}{RT}$$

The value of activation energy for each conversion is calculated from the slope of the linear plot of ln(dα/dt) versus 1/T curves.

The methods considered differ in their mathematical formulations and sensitivity to experimental data. The FR method, being differential, is more sensitive to noise but allows detailed resolution of activation energy variations. In contrast, FWO, KAS, and STK are integral methods, making them less affected by data fluctuations, with KAS and STK offering improved accuracy through refined approximations. The use of multiple approaches ensures a more robust and reliable kinetic analysis.

### 2.4. Fourier-Transform Infrared Spectroscopy (FT-IR)

FT-IR spectra (4 cm^−1^ resolution, wavenumber range 500–4000 cm^−1^) were recorded with a Shimadzu IRPrestige-21 spectrometer (Tokyo, Japan), using the ATR method. Small pieces of the PVA and PVA/glycerol films treated for 30 min at 150, 200, and 250 °C were placed in the instrument’s sample holder. The data were acquired using the software Shimadzu IR solution 1.21 (Tokyo, Japan) and analyzed using Originpro 2024 (Northampton, MA, USA) software.

## 3. Results and Discussion

**Figure 1:**
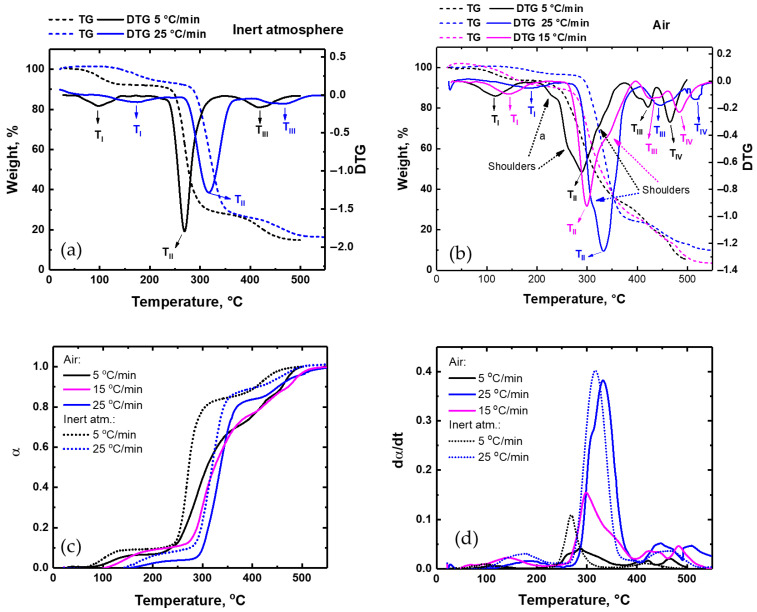
TGA and DTG curves of PVA films measured in inert atmosphere (**a**) and in air (**b**) at 5 °C/min and 25 °C/min heating rates. Calculated conversion (**c**) and reaction rate (**d**) for corresponding TGA data (dashed curves) in (**a**,**b**).

Thermogravimetric analysis was conducted on pure PVA films in air and inert atmospheres at different heating rates: 5, 15, and 25 °C/min. Figure 1a,b show the thermogravimetric curves (mass loss versus temperature, TGA) and their DTG curves for the PVA films in different atmospheres. Each peak of the DTG curves represents a separate event and corresponds to the temperature of the maximum rate of mass loss (TGA curve) [9]. The different DTG peaks have been labeled in Figure 1 with a capital T, with subscripts in capital Roman numerals. The calculated conversion (α) and conversion rate (dα/dt) corresponding to the TGA data are displayed in Figure 1c,d, respectively.

As shown in Figure 1a, the thermal transformations of the pure PVA film in an inert atmosphere occur in three main stages, which is in accordance with the literature [14,15]. It has been reported that an early weight loss process is attributed to the evaporation of non-bound and bound water molecules. The second stage involves the elimination of water from the individual PVA chains (leading to polyene formation), accompanied by random chain scissions to release acetaldehyde, saturated and unsaturated aldehydes, ketones, and some volatiles. In the third stage, the residues further undergo intermolecular cyclization to produce volatile gases, benzene, and carbonaceous material [14].

The degradation process in an air atmosphere shows some differences from that of an inert environment. As is apparent from Figure 1b, the shape of the TGA curves changes and the rate of mass loss reduces. The main degradation peak (T_II_) becomes broader in comparison with that of an inert atmosphere, especially for lower heating rates. An additional degradation stage is apparent in the DTG curves, with the thermal degradation now extending to four main stages, in accordance with [16,17]. The first stage of mass loss for PVA films in air, similarly to the inert atmosphere, is related to the loss of non-bound and bound water. The second stage refers to the partial dehydration of PVA, which is accompanied by polyene formation. In [14] it was shown that, during this step, the formation of carbonyl (C=O) groups takes place due to oxidation processes. In this work we have shown that this second degradation peak (T_II_) is severely affected by changes in the atmosphere and heating rate. Figure 1b shows that this peak is much broader in air at 5 and 15 °C/min heating rates, becoming narrower at a 25 °C/min heating rate, which strongly suggests that the peak broadening is caused by the impact of oxidation processes. The third degradation stage is related to polyenes’ decomposition to form macroradicals, acetaldehyde, benzaldehyde, acrolein, and polyconjugated aromatic structures as a result of intramolecular cyclization and condensation, while the fourth stage is attributed to the thermo-oxidation of carbonized residues [16,17]. Overall, the thermal degradation of PVA in air proceeds through a sequence of dehydration, oxidative chain scission, and eventual mineralization to low-molecular-weight products. It should be noted, however, that the detailed degradation pathways are likely to be complex and influenced by both external conditions and detailed chemical structure [18,19].

The conversion curves for pure PVA (Figure 1c) confirm that thermal decomposition under an inert atmosphere occurs rapidly, while, in air, degradation extends over broader temperature ranges due to oxidative reactions. At higher heating rates, degradation in air resembles that in an inert atmosphere. Further analysis of the reaction rate (dα/dt) curves for pure PVA (Figure 1d) illustrates that the second degradation stage, with the peak at T_II_ (as identified from DTG analysis), corresponds to the most intense variation in reaction rate. Under an inert atmosphere, the degradation peaks are sharper, higher, and narrower, compared to those in air. The reaction rate increases with the heating rate; the curves for 15 °C/min rate occupy intermediate positions between those for 5 °C/min and 25 °C/min. The difference in reaction rates for the second degradation step between inert and air atmospheres diminishes at higher heating rates, where thermal decomposition dominates over oxidative effects. In air, the low and broad degradation peak at 5 °C/min reflects the influence of oxidation. At 25 °C/min, the peak shape, sharpness, height, and width in air closely resemble those in an inert atmosphere, although the air peak remains slightly lower and broader. This indicates that oxidative effects are minimized at higher heating rates, with decomposition governed primarily by temperature.

**Figure 2:**
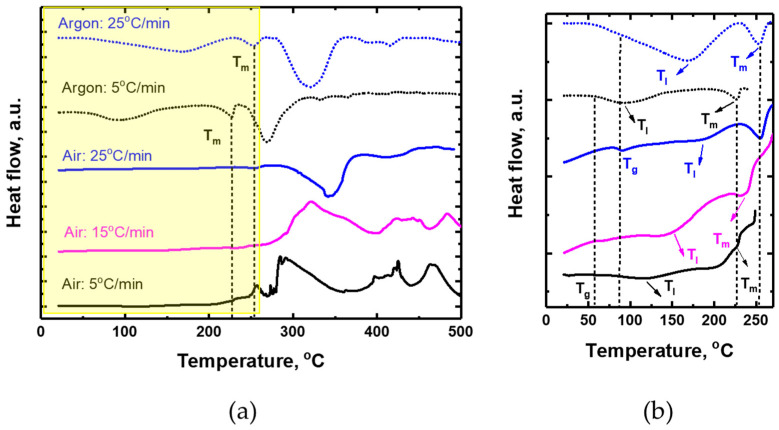
(**a**) DTA curves for pure PVA films at the heating rates of 5, 15, and 25 °C/min in air and argon atmospheres.The dashed lines indicate the position of the melting point (T_m_) at heating rates of 5 °C/min and 25 °C/min in an argon atmosphere. (**b**) Enlarged image of the corresponding region in (**a**). The additional dashed lines indicate the position of the glass transition temperature (T_g_) in air at heating rates of 5 °C/min and 25 °C/min.

At the lower heating rates (5 and 15 °C/min), the DTA curves for pure PVA in an air atmosphere (Figure 2) indicate the emergence of exothermic maxima within the temperature range corresponding to T_II_. However, those exothermic peaks are absent in the DTA curve for the same film in an inert atmosphere, or at a higher heating rate (25 °C/min) under air (see Figure 2a). These results suggest that the exothermic peaks originate from oxidation processes. The fact that the degradation in air occurs over a broader temperature range may also be attributed to additional oxidative reactions. In contrast, degradation in an inert atmosphere, in the absence of oxygen, is limited to thermal decomposition, resulting in lower final degradation temperatures for the second degradation stage at the same heating rate (see DTA data: dashed curves in Figure 1a,b). The peak decomposition temperatures under an inert atmosphere are also shifted to lower values than under air at the same heating rate (see DTG: solid curves in Figure 1a,b). Degradation in air results in a lower residue weight due to additional oxidative reactions.

Focusing on Figure 2b, on the DTA curves for pure PVA, a minimum at 57 °C can be observed for the 5 °C/min heating rate, which appears at 68 and 90 °C for the 15 and 25 °C/min heating rates, respectively, and is not accompanied by mass losses or changes in the DTG curves in either case. We attribute this peak, labeled T_g_ in Figure 2b, to the PVA glass transition temperature [20,21]. In addition, in an inert atmosphere, endothermic peaks appear at approximately 227 °C and 254 °C for heating rates of 5 and 25 °C/min, respectively. These peaks, labeled T_m_, can be attributed to the melting point of pure PVA [22,23]. For PVA films in air at a heating rate of 25 °C/min, the melting point also appears at the same temperature as under an inert atmosphere. Although the DTA curves at heating rates of 5 and 15 °C/min in air are more complex due to the overlap of melting with the onset of an exothermic process above 200 °C, the melting points appear to be at ~227 °C and ~240 °C, respectively. T_I_ determined from the DTG curves (Figure 1a,b) corresponds to endothermic minima in the DTA curves.

In Figure 1b, the DTG curves in air show several shoulders (see arrows in Figure 1b), which can be caused by different overlapping processes during degradation. At the lowest heating rate (5 °C/min), at 210–230 °C, there is a shoulder, labelled “a” in the Figure, which lies within the melting range and also coincides with the onset of the exothermic event in the DTA curve (solid black line, Figure 2b). Thomas et al. [14] observed similar shoulders in both air and inert atmospheres at 1 °C/min, concluding that they result either from inhibition of degradation due to the melting of crystallites or from a shift in the degradation mechanism. Degradation is initiated in the amorphous regions and does not extend to the crystalline regions before melting. In our case, under an inert atmosphere, this shoulder is barely noticeable, and we cannot rule out the possibility that it emerges as a result of oxidation processes in air.

The weight loss and the main characteristic temperatures of PVA degradation in air and under an inert atmosphere are presented in Table 1.

As shown in Table 1, for both atmospheres, higher heating rates (25 °C/min) shift the decomposition to higher onset, peak, and final temperatures. This is expected, as less time is available for the system to reach thermal equilibrium. Comparing the onset and final degradation temperatures at the same heating rate but under different atmospheres reveals that the temperature ranges for each stage are broader in air.

The second degradation step (with peak T_II_) is the main degradation step under all studied conditions, characterized by the highest weight loss, which increases with the heating rate (from 5 to 15 and 25 °C/min). At a slower heating rate (5 °C/min), the sample spends more time at each temperature, allowing for more gradual decomposition and partial stabilization of intermediate degradation products. These intermediates might react to form non-volatile, carbonaceous residues, reducing the overall weight loss during the main degradation step. At a higher heating rate (25 °C/min), there is less time for stabilization processes, resulting in greater breakdown of the polymer into volatile products. The release of volatiles before secondary reactions (e.g., char formation) leads to increased weight loss. This explains why under an inert atmosphere, the increase in weight loss with higher heating rates is around 3%, whereas in air it is substantially greater, reaching approximately 20%.

In the third and fourth steps, the weight loss decreases with temperature due to increased degradation in the previous step.

It must be emphasized that the balance between thermal decomposition and oxidative reactions in air is influenced by the heating rate. At 5 °C/min, oxidative reactions can proceed more fully but are moderated by the slower rate of thermal decomposition. In contrast, at 25 °C/min, rapid thermal decomposition overwhelms oxidative stabilization mechanisms, resulting in the formation of more volatile products and increased weight loss.

**Figure 3:**
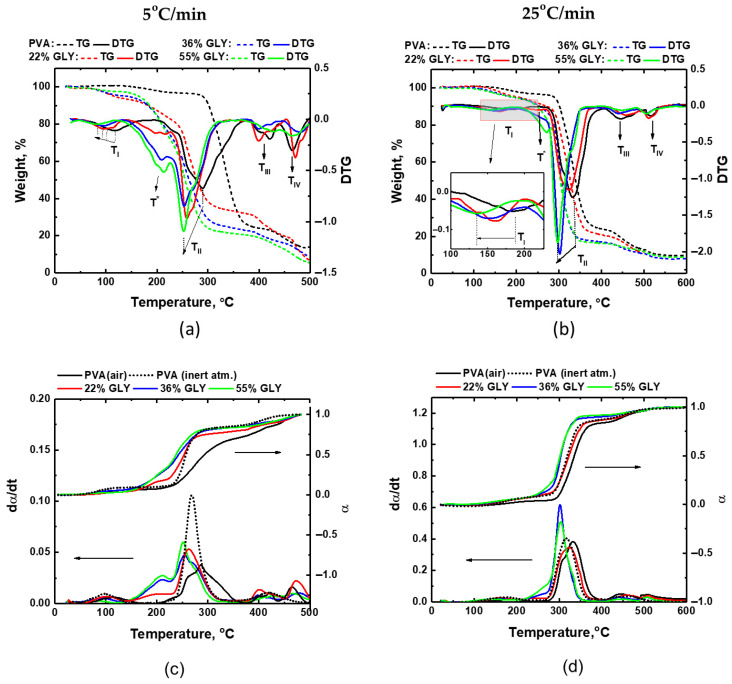
TGA and DTG curves for PVA and PVA/glycerol films measured at 5 °C/min (**a**) and 25 °C/min (**b**) heating rates in air. Calculated conversion and reaction rate at 5 °C/min (**c**) and 25 °C/min (**d**) heating rates for corresponding TGA data in (**a**,**b**) and PVA film in an inert atmosphere. In (**c**,**d**) the arrows indicate the corresponding y-axis for each set of curves.

Figure 3a,b illustrates the thermal degradation behavior of PVA films with varying glycerol contents at two heating rates in air. The calculated conversion (α) and reaction rate (dα/dt) (Figure 3c,d, respectively) are also included.

From the TGA curves in Figure 3a,b, it is apparent that the addition of glycerol reduces the thermal stability of the blends, as evidenced by the earlier onset of mass loss and the shift in the curves to lower temperatures. Also, the slope of the TGA curves at the main degradation stage becomes steeper with the increase in glycerol content. This effect is more pronounced at lower heating rates. While the DTG curves retain peaks characteristic of pure PVA, the main degradation peak shifts to lower temperatures with increasing glycerol content, accompanied by changes in peak shape and intensity. Additional peaks are observed, likely related to glycerol degradation.

Glycerol degradation is expected to contribute to the lower-temperature peaks (200–250 °C at 5 °C/min and 250–300 °C at 25 °C/min), [24], while PVA degradation dominates the higher-temperature range. This suggests that glycerol and PVA degrade independently to some extent but may also form complexes that influence the overall degradation mechanism. The overlapping and broadening of DTG peaks with the addition of glycerol indicate synergistic effects between glycerol and PVA, potentially due to plasticization or molecular interactions.

The heating rate affects the degradation behavior of PVA/glycerol blends similarly to pure PVA films. Increasing the heating rate shifts the TGA and DTG curves toward higher temperatures (Figure 3a,b). This is a typical thermal analysis phenomenon, where higher heating rates reduce the residence time for thermal decomposition, thereby requiring higher temperatures to initiate degradation. At 5 °C/min, degradation is more gradual, with broader peaks. At 25 °C/min, the DTG peaks become sharper and more defined, indicating a faster and more abrupt degradation process.

**Figure 4:**
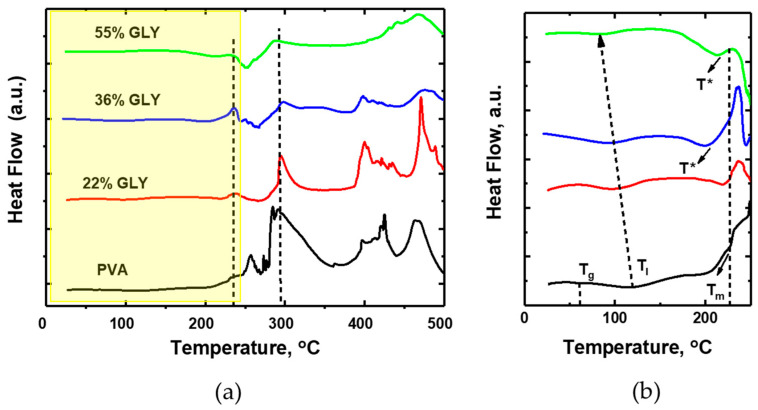
(**a**) DTA curves of PVA and PVA/glycerol films with 22% *w*/*w*, 36% *w*/*w*, and 55% *w*/*w* glycerol at a heating rate of 5 °C/min. The dashed line is intended as a visual guide to facilitate the comparison of peak positions across the different curves. (**b**) Enlarged image of the corresponding region in (**a**) The dashed lines indicate the positions of Tg and Tm for PVA, and of TI for the different samples.

Analogously to pure PVA, the first degradation stage of PVA/glycerol films is presumably related to the removal of adsorbed water. The position of the T_I_ peak shifts to lower temperatures when increasing the glycerol concentration (Figure 4). Remarkably, for the PVA/glycerol films, an additional thermal process corresponding to the DTG shoulder/peak labeled T* can be distinguished when heating in the range of 207–214 °C at a rate of 5 °C/min (Figure 3a). For the higher heating rate of 25 °C/min, this peak becomes more prominent at higher glycerol concentrations and shifts to 250–280 °C (Figure 3b). This peak is absent in pure PVA films. As previously mentioned, according to [24], the thermal degradation of pure glycerol in air is characterized by a single event occurring between 194 and 246 °C at a heating rate of 10 °C/min. Therefore, the T* peak occurring between 207 and 214 °C (250 and 280 °C) on the DTG curves of the PVA/glycerol films could be related to glycerol decomposition or degradation of glycerol-rich complexes. The second degradation stage (with the peak T_II_), characterized by the largest weight loss in pure PVA, shifts to lower temperatures with increasing glycerol content. In contrast, the positions of the third and fourth degradation peaks are largely unaffected by the addition of glycerol.

For PVA/glycerol films (Figure 3c,d), the addition of glycerol results in a steeper rise in conversion, similar to the PVA’s behavior under an inert atmosphere. This effect is more pronounced at lower heating rates, suggesting that glycerol suppresses oxidative processes and facilitates decomposition. Beyond 36% *w*/*w* glycerol content, further increases in glycerol concentration have little effect on the conversion behavior, indicating a saturation point in its influence on thermal decomposition.

Increasing the glycerol content amplifies the degradation rate (see Figure 3c,d), particularly in the temperature ranges of about 150–320 °C (for 5 °C/min) and 220–370 °C (for 25 °C/min). As the glycerol content increases, the rate of glycerol degradation—corresponding to the peak T* in DTG, at ~200 °C and ~250 °C for 5 °C/min and 25 °C/min heating rates, respectively—increases because a greater amount of glycerol is available to undergo thermal degradation. At 5 °C/min the rate of glycerol degradation reaches nearly 50% of the maximum rate of the main degradation step, while at 25 °C/min the rate of glycerol degradation is much lower than the rate of the main degradation step, corresponding to the DTG peak T_II_. This reduction at higher heating rates may result from insufficient time for complete glycerol degradation, and the rest of the glycerol degrades in the next step, overlapping with further degradation processes and increasing the rate.

At the same time, for higher glycerol contents, the rate for the main degradation step increases substantially, becoming even higher than for PVA in an inert atmosphere at a 25 °C/min heating rate.

The dα/dt curves for PVA/glycerol confirm that thermal decomposition in the presence of glycerol is rapid, with narrower peaks, indicating that oxidative processes are less dominant in the presence of glycerol. The main degradation step is composed of overlapping events, likely related to the degradation of PVA/glycerol complexes and PVA itself, and exhibits an increased reaction rate with higher glycerol content. Notably, with increasing glycerol content, the maximum rate shifts to lower temperatures. For PVA/glycerol films with 22% glycerol, the main degradation peak closely resembles that of PVA under an inert atmosphere, although the reaction rate is slightly lower, reflecting the presence of residual oxidative processes. As the glycerol content increases to 36% and 55%, the main degradation peak becomes sharper and shifts further to lower temperatures. These changes highlight enhanced interactions between PVA and glycerol, such as the formation of PVA/glycerol complexes.

The increased reaction rate suggests that glycerol actively participates in decomposition reactions, enhancing the overall thermal response. At 5 °C/min, the degradation rates for PVA/glycerol films remain lower than those for PVA under an inert atmosphere, whereas at 25 °C/min, films with higher glycerol content exhibit degradation rates exceeding those of PVA under inert conditions. This shift highlights the complex interplay of processes governing degradation. Oxidation is suppressed as the heating rate increases, while the interaction between PVA and glycerol significantly influences the degradation pathway. At lower glycerol contents, the plasticization effect enhances chain mobility and facilitates decomposition. As the glycerol content increases further, the formation of glycerol–PVA complexes or glycerol aggregates may alter the degradation pathway and rate. At higher heating rates, thermal degradation processes dominate over oxidation. The variability in degradation rates across different glycerol contents and heating rates reflects a balance between enhanced chain mobility and structural changes from glycerol–PVA interactions. These competing factors result in non-linear trends, where the degradation behavior depends on the combined influence of glycerol content and heating rate. Overall, the acceleration of degradation in PVA/glycerol films can be attributed to the plasticizing effect of glycerol, which increases PVA chain mobility, reduces the energy barrier for decomposition reactions, and enables these reactions to proceed more efficiently.

Figure 4 shows the DTA curves of PVA and PVA/glycerol films in air at a heating rate of 5 °C/min. The DTA curves for PVA/glycerol films at a higher heating rate (25 °C/min) were discussed in our previous paper (Figure 4 from Kovtun et al. [8]).

The DGT peak T_II,_ as observed in Figure 3a at around 289 °C, can be correlated with the exothermic peak highlighted by a dashed line in Figure 4a, which is consistent with the occurrence of oxidation processes. In DTA, this peak also appears for the film containing 22% *w*/*w* glycerol, albeit narrower and lower in intensity. For higher glycerol concentrations (36% *w*/*w* and 55% *w*/*w*) the peak is only faintly visible. For the 22% *w*/*w* glycerol film, another small exothermic reaction is evident in the DTA curves at around 235 °C (highlighted by a dashed line in Figure 4a). This peak increases slightly for the 36% *w*/*w* glycerol film but becomes barely detectable in the 55% *w*/*w* glycerol film. Notably, this exothermic reaction appears to be followed by an endothermic event as the temperature increases. We tentatively attribute this exothermic reaction to the incipient oxidation of PVA–glycerol complexes. As discussed previously, the presence of glycerol may alter the reaction pathways, restraining the oxidation process, and favoring thermal decomposition instead.

The DTG peak T* observed in Figure 3a at around 210 °C, attributed to the degradation of glycerol, can be correlated with the endothermic DTA peak indicated by an arrow in Figure 4b. This peak is apparent for the 36% *w*/*w* glycerol film, but it cannot be observed for the 22% *w*/*w* glycerol film. As a matter of fact, in the DTG curves, the contribution of the T* component is much smaller for this film. For the 55% *w*/*w* glycerol film, a DTA endothermic peak possibly related to T* is also evident in Figure 4b, shifted to higher temperatures.

According to our previous investigation [8], based on FT-IR, XRD, and advanced SPM data, when incorporated into semicrystalline PVA in small quantities, the glycerol molecules primarily interact with the PVA chains through hydrogen bonds formed by their secondary alcohol groups. Glycerol does not penetrate the PVA crystallites but promotes the reorganization of the amorphous PVA regions, alters the shape of the PVA crystalline domains, and preferentially accumulates at the amorphous/crystalline interface. As the glycerol content increases, glycerol aggregates form and begin to penetrate the PVA crystalline structures, leading to their disruption at high glycerol contents. Hence, the appearance of a significant T* component is actually not expected for the film with 22% *w*/*w* glycerol content, in which most of the added glycerol is incorporated as individual molecules to the PVA chains, and the PVA would maintain (or even slightly increase) its crystallinity. In contrast, the structure of the 55% *w*/*w* glycerol film is consistent, with the presence of glycerol aggregates in a disrupted amorphous PVA matrix.

In Figure 4b, the melting point Tm of the pure PVA sample (black curve) is also noticeable as an endothermic minimum, highlighted by an arrow. The film with 22% *w*/*w* glycerol also contains PVA crystalline domains. A slight displacement of the melting point Tm to a lower temperature in this case might be explained by the presence of glycerol at the PVA crystalline domain interface.

Eventually, the first DTA endothermic minimum T_I_ in Figure 4b is well correlated with the DTG T_I_ peak in Figure 3a and shifts to lower temperatures with an increase in glycerol content.

**Figure 5:**
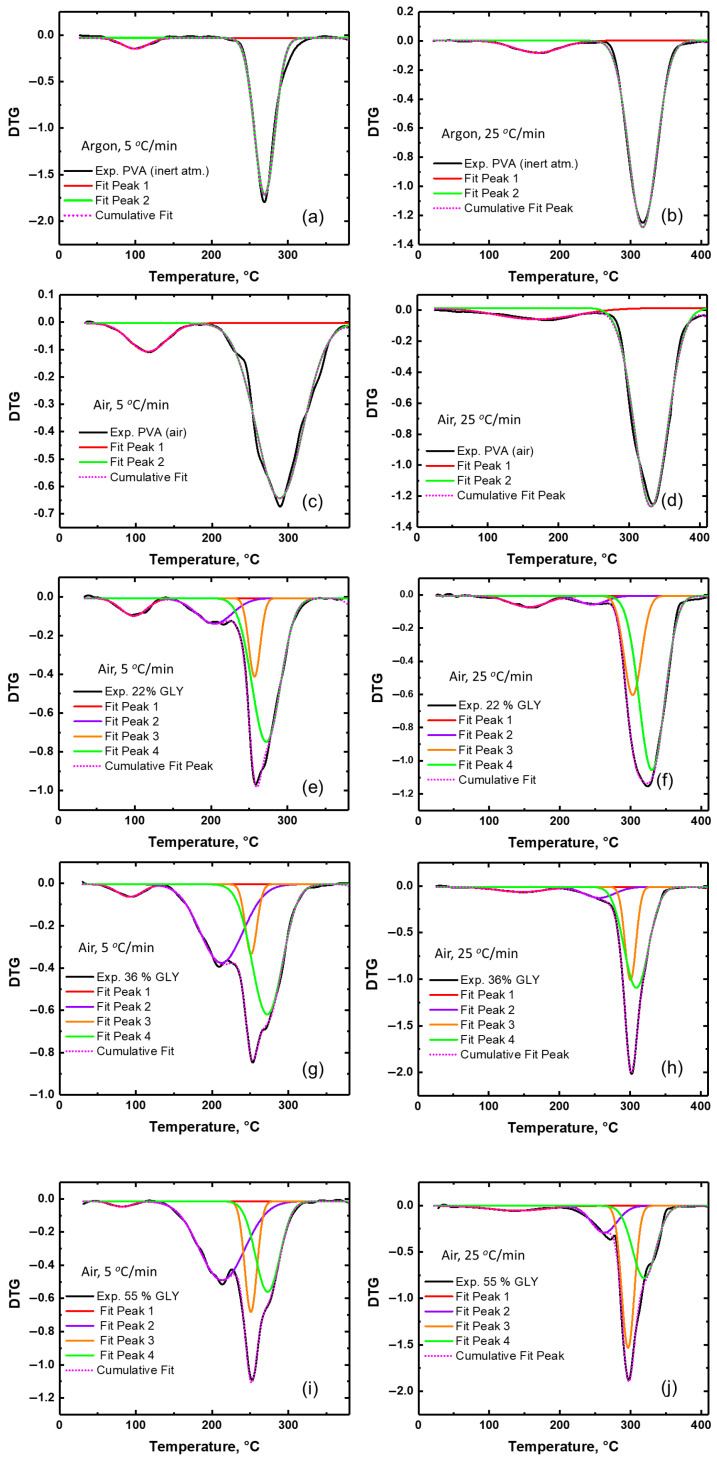
Peak deconvolution results for DTG curves for pure PVA in an inert atmosphere (**a**,**b**); pure PVA in air (**c**,**d**); PVA/glycerol films in air (**e**–**j**) measured at heating rates of 5 °C/min (**e**,**g**,**i**) and 25 °C/min (**f**,**h**,**j**).

To analyze the possibility of overlapping processes in thermal decomposition, deconvolution of DTG curves was performed. Different functions can be used for deconvolution analysis: symmetric functions such as the Gaussian and Lorentz functions, and the asymmetric Weibull or Fraser–Suzuki functions [25,26]. While the asymmetric functions are considered to be more suitable for deconvolution procedures in TGA [26], the symmetric functions might provide a reasonable approximation and can be used for preliminary or qualitative analysis. In our case, Gaussian fitting was used, considering the minimum number of component peaks corresponding to the characteristic degradation peaks. Additional peaks were introduced only when the fitting was unsuccessful. The high correlation coefficients (R^2^ > 0.95) between experimental data and the multi-peak fitting confirm the reliability of the deconvolution. Figure 5 shows the results of DTG peak deconvolution for pure PVA films in an inert atmosphere (Figure 5a,b) and in air (Figure 5c,d), as well as for PVA/glycerol films in air at two heating rates (5 °C/min and 25 °C/min; Figure 5e–j). For pure PVA films, the curves are well described by the fitting procedure. However, the asymmetric shape of some peaks suggests that alternative asymmetric functions, such as the Fraser–Suzuki and Weibull functions [26], might improve accuracy in future analyses. Although the main degradation peak for PVA in air has been described by a single Gaussian peak, the slight indication of shoulders in the peak shape and its broader nature compared to that in an inert atmosphere likely reflect contributions of different mechanisms (see the aforementioned discussion in relation to Figure 1, just before the introduction of Table 1). Nevertheless, additional component peaks were not introduced, so as to avoid unnecessary complexity in the fitting.

For PVA/glycerol films, the DTG deconvolution reveals a more complex degradation profile compared to that of pure PVA (Figure 5e–j). The emergence of a shoulder in the DTG curve, which transforms into a distinct minor peak at higher glycerol concentrations, is attributed to a glycerol degradation peak (peak 2 in Figure 5e–j). This feature becomes more pronounced at lower heating rates, reflecting the enhanced contribution of glycerol to the thermal decomposition process. The main degradation peak of PVA/glycerol films (T_II_) is no longer described by a single peak, as seen in pure PVA, but rather by two overlapping peaks. For the film with the lowest glycerol content (Figure 5e,f), the first of these overlapping peaks (peak 3) is located at about 250 °C for 5 °C/min and 300 °C for 25 °C/min, and it may be attributed to the degradation of PVA/glycerol complexes. The second (peak 4) is located at about 272 °C for 5 °C/min and 320 °C for 25 °C/min. The degradation peak T_II_ for pure PVA in an inert atmosphere is at 269 °C for 5 °C/min and 317 °C for 25 °C/min. Hence, the deconvoluted peak 4 for PVA/glycerol films likely corresponds to the degradation of PVA. The fact that the position of peak 4 for PVA/glycerol films corresponds to that of PVA degradation in an inert atmosphere, rather than in air, is possibly due to the suppression of oxidation processes in the presence of glycerol. The shape of peak T_II_ changes with the addition of glycerol, becoming narrower. With increasing glycerol concentration, the relative intensity of peak 3 increases, indicating an enhanced contribution of PVA/glycerol interactions to the degradation process. Furthermore, the overlap between peaks 2 (glycerol) and 3 (PVA/glycerol complexes) becomes more significant, emphasizing the role of glycerol in modifying the thermal behavior of the blends. Table 2 shows the values of peak heights and full width at half-maximum (FWHM) of the deconvoluted peaks.

**Figure 6:**
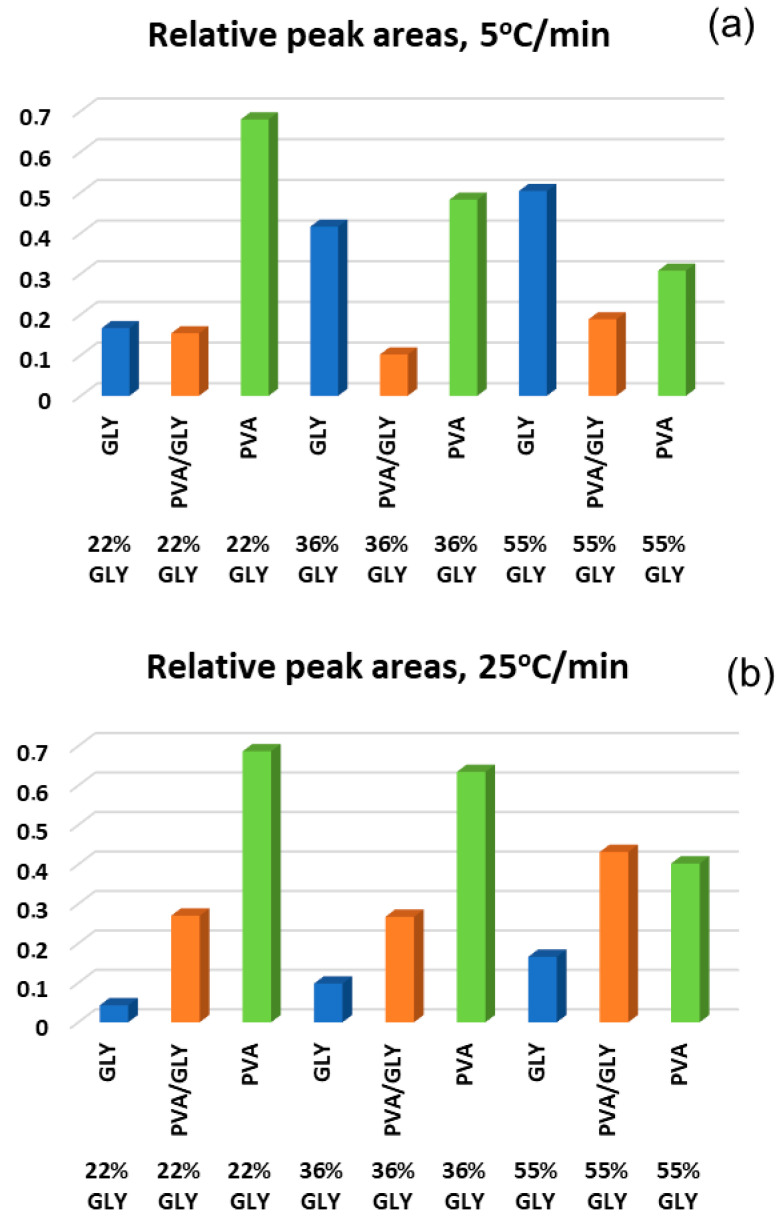
Ratio of the areas of deconvoluted peaks from Figure 5e–j for glycerol (peak 2), PVA/glycerol (peak 3), and pure PVA (peak 4) for PVA/glycerol films at 5 °C/min (**a**) and 25 °C/min (**b**) heating rates.

Figure 6 shows the relative peak areas of the deconvoluted peaks presented in Figure 5. The area under each peak was divided by the total area of the three deconvoluted peaks (peaks 2–4). The three deconvoluted peaks correspond to thermal events associated with glycerol decomposition (peak 2 of Figure 5e–j), PVA/glycerol complex degradation (peak 3 of Figure 5e–j), and PVA degradation (peak 4 of Figure 5e–j). At a 5 °C/min heating rate, at lower glycerol content (22% *w*/*w*), the relative contribution of PVA degradation is dominant, with low influence from glycerol or PVA/glycerol complexes. This is consistent with the manner in which glycerol incorporates to the PVA matrix, according to the results reported in [8] (see related discussion above in the context of Figure 4).

With increasing glycerol content (36% *w*/*w* and 55% *w*/*w*), the peak contribution from glycerol decomposition increases, while the contribution from PVA degradation decreases, indicating a shift in the thermal degradation profile toward glycerol-dominated processes.

At the higher heating rate, the trends are similar; however, the contribution of glycerol decomposition is significantly less pronounced across all glycerol contents, particularly for the 36% *w*/*w* and 55% *w*/*w* glycerol films. The degradation of PVA/glycerol exhibits a higher relative contribution at 25 °C/min compared to 5 °C/min, suggesting that incomplete degradation of glycerol at the higher heating rate allows the remaining glycerol to further interact with PVA and contribute to the degradation of PVA/glycerol complexes. The contribution of PVA degradation remains significant at 22% glycerol but decreases notably for higher glycerol contents.

Stepwise isothermal degradation experiments at different temperatures (i.e., 50, 150, 200, 250, and 300 °C) were conducted to gain further insight into the time- and temperature-dependent processes occurring during PVA and PVA/glycerol degradation.

**Figure 7:**
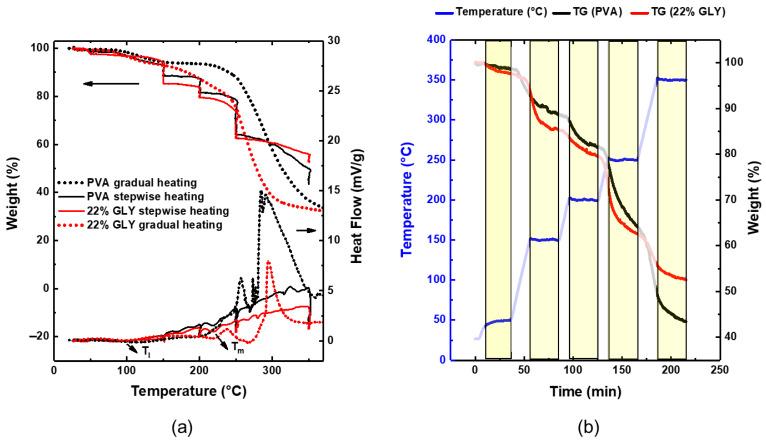
(**a**) Weight loss and heat flow as functions of temperature for PVA (black) and PVA/22% glycerol (red) during stepwise isothermal degradation in air (solid curves), and during continuous heating at 5 °C/min (dotted curves). The horizonal arrows indicate the corresponding y-axis for each set of curves (**b**) Weight loss and temperature as functions of time for PVA (black) and PVA/22% glycerol (red) during stepwise isothermal degradation in air.

Figure 7 summarizes the main findings of this study. Figure 7a shows a comparison of the weight loss and heat flow as functions of temperature for pure PVA and PVA/22% glycerol, obtained using two heating modes: stepwise isothermal heating and gradual heating. Figure 7b shows the weight loss for the same samples as a function of time, with isothermal plateaus highlighted in yellow.

At temperatures below 150 °C, no significant difference was observed between stepwise and gradual thermal degradation, indicating that the primary decomposition reactions had not yet begun, and that the heating mode (stepwise vs. gradual) had minimal impact. The weight loss for PVA/glycerol in this region was slightly higher (Table 3), presumably due to its higher water content.

At 150 °C, pure PVA exhibits notable weight loss (Figure 7a,b, Table 3) and an initial increase in heat flow under stepwise heating, in contrast to the minimal changes observed during gradual heating (Figure 7a). At this stage, it is likely that any remaining absorbed water has already evaporated. Therefore, the observed changes may be attributed to the onset of early-stage oxidation reactions, which likely require a longer residence time or higher temperatures to proceed significantly. For PVA/22% glycerol, the weight loss at 150 °C is greater and exhibits a much steeper time dependence (Figure 7b). This is likely due to glycerol degradation rather than oxidation of the PVA matrix itself.

At the next isothermal steps (200 and 250 °C), the weight loss becomes significantly greater during stepwise heating than during gradual heating (see Figure 7a), particularly for pure PVA (Table 3). The kinetics profiles within the isothermal plateaus (Figure 7b) exhibit steeper weight loss for pure PVA compared to the glycerol-containing films. This suggests that during those isothermal heating stages, the degradation pathways of PVA are altered, resulting in higher degradation rates for pure PVA compared to the PVA/glycerol films. Remarkably, the difference in weight loss between gradual and stepwise heating is less pronounced in the glycerol-containing samples (see Figure 1a), indicating that their degradation behavior is less affected by the heating profile.

At 200 °C, while the pure PVA sample is expected to be at the onset of its main degradation event, in the PVA/glycerol samples the degradation of PVA/glycerol complexes is likely to occur preferentially over that of the pure PVA phases (see Figure 5 of the revised manuscript). It should be noted that the results discussed here correspond to the PVA/glycerol sample with 22% glycerol content—the lowest among the tested compositions.

At 350 °C, the weight loss during the isothermal step is lower than during the previous isothermal plateau (250 °C) and significantly lower than under gradual heating. The mass loss at this stage may result from processes that were already activated and partially completed during earlier stepwise holds, as well as from the incomplete decomposition of more stable carbonaceous residues. At this stage, the PVA/glycerol sample also shows less degradation than pure PVA.

For temperatures around 300 °C, the heat flow behavior during gradual heating reveals a sharp exothermic peak, typical of rapid oxidative decomposition (Figure 1a, dotted curves). In contrast, stepwise heating shows a gradual increase in heat flow without distinct maxima (solid curves), indicating slower but more continuous oxidation across the temperature range. This effect is particularly evident for pure PVA. For the PVA/glycerol film, the heat flow also increases progressively during stepwise heating, but with a lower slope, indicative of a more stable (less exothermic) degradation pathway.

The faster degradation observed during stepwise heating in the temperature range of 150–250 °C—particularly for the PVA sample—can be attributed to kinetic limitations under gradual heating. Although oxidation may initiate at lower temperatures, the reaction rate is slow; thus, gradual heating does not allow it to complete before the temperature increases further. As a result, oxidation is delayed and appears to occur at higher temperatures under gradual heating. In contrast, under the stepwise heating mode, other thermal degradation processes competitive with oxidative processes may also take place, leading to greater mass loss and a smoother, more distributed increase in heat flow. The results confirm that oxidative degradation of PVA is highly time- and temperature-dependent.

**Figure 8:**
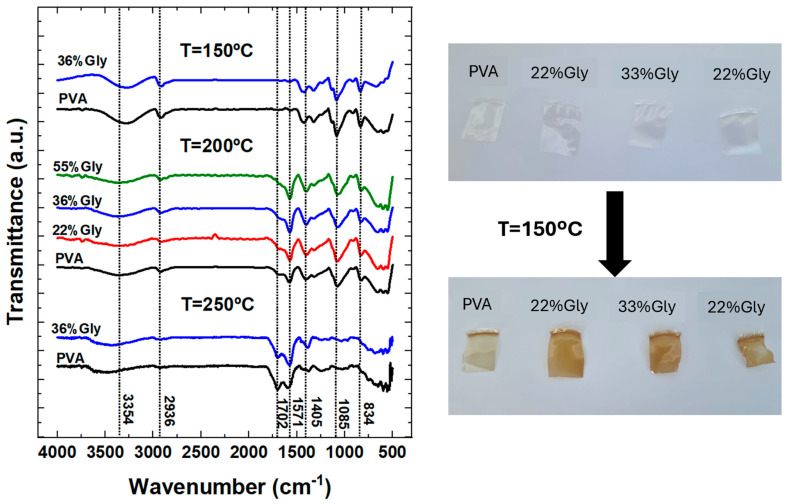
FT-IR of PVA and PVA/Gly after isothermal treatment at 150 °C, 200 °C, and 250 °C for 30 min. Photos: untreated films (upper row) and films after treatment at 150 °C (lower row).

To better understand the reactions accompanying the thermal degradation process, we conducted isothermal heating of pure PVA and PVA/glycerol films at 150 °C, 200 °C, and 250 °C in a conventional oven, following a procedure analogous to that used for stepwise isothermal degradation. Each temperature was maintained for 30 min, and the treated samples were subsequently analyzed using FT-IR. The maximum allowed temperature deviation was set to ±5 °C, and the oven took approximately 45 min to stabilize from room temperature to the selected temperature, with the samples kept inside throughout the entire period. The results of these experiments are presented in Figure 8.

The untreated samples were completely transparent (see photos: Figure 8, right-hand side, upper row), and the plasticizing effect of glycerol—more noticeable with increasing glycerol content—could be felt by touch. After the isothermal treatments, changes in color and shape, dependent on the film’s glycerol content, became apparent. The photos in the lower row (right-hand side) show the samples after treatment at 150 °C. As seen in the images, the higher the glycerol content, the more intense the acquired brownish coloration and the greater the distortion in shape. This shape distortion was primarily observed in samples with higher glycerol concentrations of 36% and 55%.

The brownish coloration observed in heated PVA films primarily originates from the formation of conjugated polyene sequences along the polymer backbone during thermal dehydration and degradation [18], which absorb visible light. More conjugation yields a darker, more brownish appearance. The fact that the samples with higher Gly content exhibit more intense coloration may be attributed to a higher presence of conjugated species. The shape distortion observed in the samples with higher glycerol concentrations can be attributed to glycerol degradation, which occurs at lower temperatures. Voids or microcavities likely form in regions where glycerol was initially present, leading to the development of internal stresses that, in turn, result in shape distortion.

The FT-IR results of the untreated samples were presented in Figure 2 of reference [8].

From Figure 8, it is apparent that after treatment at 150 °C, the band corresponding to (O-H) stretching vibrations—associated with intermolecular and intramolecular hydrogen bonds, and initially observed at ~3273 cm^−1^ for the untreated pure PVA and PVA/Gly films [8]—shifts to ~3290 cm^−1^. This band is further displaced to higher wavenumbers, reaching ~3357 cm^−1^ and ~3431 cm^−1^ after treatments at 200 °C and 250 °C, respectively. In addition, a considerable decrease in band intensity is observed, particularly after the 200 °C treatment. These changes may be explained by the loss of absorbed water, as well as the dehydration processes accompanying the thermal degradation of both PVA and glycerol.

A reduction in the intensity of the two bands located at 2939 and 2907 cm^−1^ in the untreated samples—corresponding to the asymmetric and symmetric stretching vibrations of methylene (–CH_2_–), respectively—is also evident, becoming more pronounced at the higher treatment temperatures of 200 °C and 250 °C. This observation confirms that the backbone of the PVA chains is being modified, possibly through the formation of polyene structures.

In the untreated samples, small peaks were observed at 1709, 1655, and 1550 cm^−1^, which were assigned to stretching vibrations of (C=O) and (C-O) bonds present in residual acetate units. As shown in Figure 8, a very small peak appears at 1571 cm^−1^ after treatment at 150 °C. After the 200 °C treatment, the intensity of this peak increases significantly, with a stronger increase observed in samples with higher glycerol content. At this temperature, a second peak also emerges at ~1702 cm^−1^. After heating at 250 °C, the peak at 1702 cm^−1^ becomes more intense than the one at 1571 cm^−1^ in the pure PVA film, while in the PVA/Gly film the 1751 cm^−1^ peak remains predominant.

The increase in intensity of the 1702 cm^−1^ peak confirms the formation of C=O groups, indicating the occurrence of oxidative reactions within the PVA matrix. Meanwhile, the enhanced band at 1571 cm^−1^ in Figure 8 may be attributed to (C-O) vibrations arising from oxidative modifications or molecular rearrangements, or possibly from (C=O) or (C=C) stretching modes associated with the formation of polyene structures.

The peaks appearing at 1417 cm^−1^ and 1327 cm^−1^ in the untreated samples—attributed to bending vibrations of hydroxyl (–OH) and wagging of (C–H), respectively—are apparently shifted to 1435 cm^−1^ and 1320 cm^−1^, respectively, after treatment at 150 °C. The first peak shifts further to ~1405 cm^−1^ after treatments at 200 °C and 250 °C, while the relative intensity of the second peak is significantly reduced, becoming nearly absent after heating at 250 °C. These results confirm that chemical and structural changes occur in the PVA molecular chains as thermal degradation progresses.

In the untreated samples, a characteristic band around 1039 cm^−1^ clearly indicates the presence of glycerol within the PVA matrix. Remarkably, this peak is absent in the spectra shown in Figure 8, presumably reflecting that glycerol degradation is already complete at 150 °C, the lowest temperature used in the heating treatments.

Isoconversional methods are among the most reliable approaches for analyzing thermoanalytical data. They allow us to evaluate the effective activation energy (Ea) without the need to assume a specific reaction model. In the case of single-step processes, the activation energy calculated with an isoconversional method remains constant with the conversion (α). However, in complex multi-step reactions or transformations, variations in the dependency of activation energy on conversion are detected and can be associated with a change in the reaction mechanism or in the rate-limiting step of the overall reaction rate [27,28].

In this study, the activation energy was estimated using isoconversional approaches based on three heating rates for pure PVA in air and two heating rates for pure PVA under an inert atmosphere and PVA/glycerol films in air. While it is recommended to use more heating rates for higher accuracy, the present study provides an approximate evaluation of the thermal degradation kinetics of the films.

Four different isoconversional methods—the Friedman (FR), Kissinger–Akahira–Sunose (KAS), Flynn–Wall–Ozawa (FWO), and Starink (STK) methods—were employed to estimate Ea and evaluate its dependence on conversion (α). The consistency of the trends observed across these methods confirms their reliability, although minor variations in absolute Ea values were noted.

**Figure 9:**
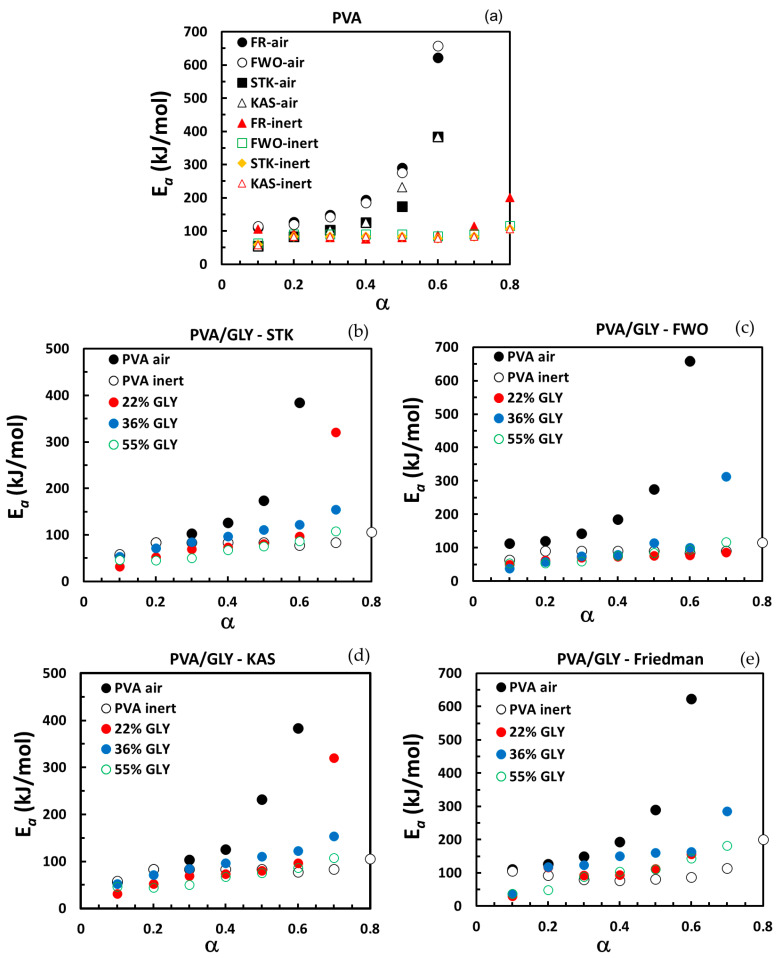
The apparent activation energy as a function of conversion for the thermal decomposition of PVA film in air and in an inert atmosphere (**a**). The apparent activation energy as a function of conversion for the thermal decomposition of PVA and PVA/glycerol films in air and PVA films in an inert atmosphere, calculated with different methods: (**b**) Starink, (**c**) Flynn–Wall–Ozawa, (**d**) Friedman, and (**e**) Kissinger–Akahira–Sunose.

For pure PVA, the calculated apparent activation energies (Figure 9a) reveal distinct behaviors in air and under inert atmospheres. In air, Ea gradually increases with conversion, indicating the multistage degradation mechanism and suggesting that thermal degradation becomes progressively more energy-demanding. This behavior is likely attributable to the oxidative environment, where degradation products may form a protective char layer or involve the formation of structures that are increasingly resistant to breakdown as conversion progresses. Such Ea(α) dependence is consistent with previous work [16], where the activation energy increased with conversion from ~100 kJ/mol to ~400 kJ/mol and from ~75 kJ/mol to ~200 kJ/mol for the Friedman and WFO methods, respectively.

In contrast, for PVA in an inert atmosphere, the Ea values remain relatively constant (~90 kJ/mol) across the main degradation step 0.2 < α < 0.7, which is consistent with the results reported by Zhao et al. [15] This stability suggests that thermal degradation under inert conditions proceeds through relatively uniform energy-requiring processes, likely involving the cleavage of similar chemical bonds. At higher conversions (α > 0.7), the increase in Ea could reflect the presence of more thermally stable residual structures, such as carbonaceous char, which require more energy to decompose.

Figure 9b–e illustrate the influence of glycerol on the dependence of the activation energy on conversion for PVA degradation in air, with a comparison to the Ea(α) dependence for the degradation of pure PVA in an inert atmosphere. At lower conversions of 0.1 < α < 0.3, the Ea values for PVA/glycerol films are very close to those for pure PVA, reflecting minimal disruption to the initial degradation mechanism. As the conversion increases (α > 0.3), the Ea values for PVA/glycerol films become noticeably lower than those for pure PVA in air. This reduction in Ea suggests that the addition of glycerol plasticizes the PVA matrix, lowering the energy barrier for bond cleavage and facilitating degradation. During the main degradation step (0.2 < α < 0.6), while the Ea for PVA/glycerol films slightly increases with α, this dependence becomes less pronounced compared to pure PVA in air, and more similar to the behavior of pure PVA in an inert atmosphere. This trend indicates that glycerol modifies the degradation process, making it more similar to conditions with reduced oxidative influence. This effect can be attributed to glycerol promoting the formation of volatile degradation products or inhibiting oxidative reactions, thus limiting the extent of oxidation-driven processes. The Ea values for PVA/glycerol films exhibit greater dependency on conversion compared to those for PVA in an inert atmosphere, indicating that residual oxidative effects may still be present under an air atmosphere. Additionally, as demonstrated by peak deconvolution, the main degradation peak for PVA/glycerol films comprises two overlapping processes. These overlapping processes likely contribute to the observed variability in Ea values, with conversion reflecting the complex interplay between the thermal degradation of PVA, glycerol, and their complexes.

The higher Ea for higher conversion (α > 0.6) may suggest that, at this stage, the remaining material is less influenced by glycerol and increasingly dominated by the PVA degradation behavior. This could indicate the presence of more stable structures that require higher energy to decompose.

The isoconversional analysis demonstrated that glycerol modifies the thermal degradation kinetics of PVA by lowering the activation energy, reducing oxidative reactions, and promoting thermally driven processes.

## 4. Conclusions

This study investigated the effects of glycerol on the thermal degradation of PVA in an air atmosphere at different heating rates.

TGA and DTA of pure PVA films in both air and inert atmospheres confirmed that oxidative conditions significantly influence degradation, particularly at lower heating rates.

Thermal analysis of the PVA/glycerol films (in air) at 5 °C/min and 25 °C/min revealed distinct shifts in degradation behavior, depending on glycerol content and heating rate.

Our study confirms that degradation onset shifts to lower temperatures with increasing glycerol content and decreasing heating rate. Deconvolution of differential thermogravimetric curves revealed an additional peak corresponding to glycerol degradation, absent in the pure PVA films. The main degradation peak for PVA/glycerol blends consists of two overlapping components, which may correspond to the degradation of PVA/glycerol complexes and to that of PVA itself, respectively. Increasing the glycerol concentration enhances the contribution of the PVA/glycerol component, indicating that glycerol influences the thermal degradation pathways of PVA.

Conversion curves have shown that the addition of glycerol results in a more abrupt increase in conversion, similar to the behavior observed for pure PVA in an inert atmosphere. The dα/dt curves highlight the role of glycerol in accelerating degradation rates, particularly in the temperature range corresponding to glycerol degradation and the main degradation peak. At higher heating rates (25 °C/min), the degradation rates within the range of the main degradation peak for PVA/glycerol films exceed those of pure PVA in an inert atmosphere, indicating a synergistic effect of glycerol.

The activation energy was estimated using isoconversional methods. Pure PVA shows an increasing Ea with conversion, indicating a complex, multi-step degradation mechanism. In contrast, glycerol-plasticized films exhibit lower Ea values and reduced dependence on conversion, making their behavior more similar to degradation under an inert atmosphere. This behavior suggests that glycerol suppresses oxidative reactions and promotes earlier thermal degradation by increasing matrix flexibility.

These findings emphasize the dual role of glycerol as a plasticizer and a modifier of thermal degradation pathways. By disrupting hydrogen bonding and increasing matrix mobility, glycerol not only accelerates thermal degradation but also suppresses oxidative reactions. These insights provide a deeper understanding of the thermal behavior of PVA/glycerol blends, which is critical for optimizing their performance in applications requiring thermal stability. Additionally, the incorporation of glycerol into PVA systems can be advantageous for sustainable applications such as thermal recycling or controlled biodegradation, where lower energy input to initiate degradation is desirable.

## Figures and Tables

**Table 1:** Characteristic temperatures of PVA degradation in air and inert atmosphere.

Sample	Degradation Step	Weight Loss, %	Onset Temp., °C	Peak Temp., °C	Final Temp., °C
5 °C/min Air	I	6.3	42.6	117	185
	II	54.8	199	289	374
	III	20	374	422	439
	IV	13.5	465	446	500
15 °C/min Air	I	9.5	79	143	216
	II	64.2	244	298	396
	III	11.8	396	425	461
	IV	8.5	461	483	520
25 °C/min Air	I	3.5	66	186	250
	II	72.8	250	333	403
	III	10.3	403	445	500
5 °C/min Inert Atmosphere	I	8.1	50	98	145
	II	63.6	198	269	347
	III	13.2	347	418	190
25 °C/min Inert Atmosphere	I	7.5	88	174	260
	II	66.3	260	317	402
	III	9.7	402	469	525

**Table 2:** The height and FWHM for deconvoluted peaks related to GLY, PVA/GLY, and PVA degradation.

Sample		5 °C/min			25 °C/min		
		GLY	PVA/GLY	PVA	GLY	PVA/GLY	PVA
22% *w*/*w* GLY	Height	−0.13	−0.40	−0.74	−0.05	−0.60	−1.05
	FWHM	54.48	17.67	43.78	45.11	29.00	42.32
36% *w*/*w* GLY	Height	−0.37	−0.33	−0.61	−0.12	−0.99	−1.08
	FWHM	66.28	17.96	46.86	51.40	18.46	40.71
55% *w*/*w* GLY	Height	−0.48	−0.67	−0.55	−0.29	−1.53	−0.78
	FWHM	70.06	18.88	37.46	42.55	21.18	38.76
PVA (inert atm.)	Height	-	-	−1.69	-	-	−1.28
	FWHM	-	-	31.15	-	-	45.79
PVA (air)	Height	-	-	−0.64	-	-	−1.28
	FWHM	-	-	71.85	-	-	54.81

**Table 3:** Weight loss for PVA and PVA/22% glycerol during stepwise isothermal heating.

Heating Range	Sample	Weight Loss, %		
		During Heating	Isothermal	Total
28–50 °C	PVA	0.6	0.8	1.4
	22% GLY	0.7	1.7	2.4
50–150 °C	PVA	5.9	4	9.9
	22% GLY	4.3	8	12.3
150–200 °C	PVA	1	5.8	6.8
	22% GLY	1.7	4.1	5.8
200–250 °C	PVA	2.8	13.7	18.5
	22% GLY	5.9	11	16.9
250–350 °C	PVA	15.1	5.6	20.7
	22% GLY	6.8	3.2	10

## Data Availability

The data presented in this study are available upon request from the corresponding authors.

## Abbreviations

The following abbreviations are used in this manuscript:

TGA	Thermogravimetric analysis
DTG	Differential thermogravimetry
DTA	Differential thermal analysis
FWHM	Full width at half-maximum
PVA	Poly(vinyl alcohol)
GLY	Glycerol
FR	Friedman
STK	Starink
FWO	Flynn–Wall–Ozawa
KAS	Kissinger–Akahira–Sunose
